# Multiplexed immunofluorescence identifies high stromal CD68^+^PD-L1^+^ macrophages as a predictor of improved survival in triple negative breast cancer

**DOI:** 10.1038/s41598-021-01116-6

**Published:** 2021-11-03

**Authors:** James Wang, Lois Browne, Iveta Slapetova, Fei Shang, Kirsty Lee, Jodi Lynch, Julia Beretov, Renee Whan, Peter H. Graham, Ewan K. A. Millar

**Affiliations:** 1 St George and Sutherland Clinical School, University of New South Wales Sydney, Kensington, Australia; 2 Cancer Care Centre, St George Hospital, Kogarah, Australia; 3 Biomedical Imaging Facility, Mark Wainwright Analytical Centre, University of New South Wales Sydney, Kensington, Australia; 4 Department of Clinical Oncology, Prince of Wales Hospital, Chinese University of Hong Kong, Shatin, Hong Kong; 5 Department of Anatomical Pathology, New South Wales Health Pathology, St George Hospital, Kogarah, Australia; 6 Faculty of Medicine and Health Sciences, Western Sydney University, Campbelltown, Australia; 7 University of Technology, Sydney, Australia

**Keywords:** Cancer, Immunology, Biomarkers, Medical research, Oncology

## Abstract

Triple negative breast cancer (TNBC) comprises 10–15% of all breast cancers and has a poor prognosis with a high risk of recurrence within 5 years. PD-L1 is an important biomarker for patient selection for immunotherapy but its cellular expression and co-localization within the tumour immune microenvironment and associated prognostic value is not well defined. We aimed to characterise the phenotypes of immune cells expressing PD-L1 and determine their association with overall survival (OS) and breast cancer-specific survival (BCSS). Using tissue microarrays from a retrospective cohort of TNBC patients from St George Hospital, Sydney (n = 244), multiplexed immunofluorescence (mIF) was used to assess staining for CD3, CD8, CD20, CD68, PD-1, PD-L1, FOXP3 and pan-cytokeratin on the Vectra Polaris™ platform and analysed using QuPath. Cox multivariate analyses showed high CD68^+^PD-L1^+^ stromal cell counts were associated with improved prognosis for OS (HR 0.56, 95% CI 0.33–0.95, p = 0.030) and BCSS (HR 0.47, 95% CI 0.25–0.88, p = 0.018) in the whole cohort and in patients receiving chemotherapy, improving incrementally upon the predictive value of PD-L1^+^ alone for BCSS. These data suggest that CD68^+^PD-L1^+^ status can provide clinically useful prognostic information to identify sub-groups of patients with good or poor prognosis and guide treatment decisions in TNBC.

## Introduction

Breast cancer is the most commonly diagnosed cancer globally and the leading cause of cancer-related death in women, with over 2,000,000 new breast cancer diagnoses and almost 685,000 deaths estimated to have occurred in 2020^1^. In Australia, the incidence has increased over the last five years to reach 20,030 projected diagnoses in 2021 with over 3000 deaths, making it the second leading cause of cancer-related death in women^2^. Triple negative breast cancer (TNBC), which lacks expression of oestrogen receptor (ER), progesterone receptor (PR), or human epidermal growth factor receptor 2 (HER2), makes up 10–15% of all diagnosed breast cancers^3^ and remains a problem aggressive disease, with a reported five-year survival rate ranging from 62 to 77%^4–9^. Several large genomics studies have demonstrated high levels of tumour heterogeneity with no identifiable actionable targets and no biomarkers in routine clinical use to guide chemotherapy, which is currently the standard systemic therapy^10–13^. Tumour infiltrating lymphocytes (TILs) are now an established biomarker for TNBC, with several key studies demonstrating that tumours with ≥ 30% stromal TILs (sTILs) treated with neoadjuvant chemotherapy have better rates of pathological complete response (pCR) and generally improved longer-term survival outcomes, even in patients who never received chemotherapy^14–18^. Programmed death-ligand 1 (PD-L1) and programmed cell death protein-1 (PD-1) targeted immunotherapy combined with chemotherapy in the IMpassion130 and KEYNOTE-355 trials have shown improved progression free survival in metastatic TNBC^19,20^. Combination neoadjuvant chemotherapy and PD-1/PD-L1 axis immunotherapy has also demonstrated treatment benefit for early-stage TNBC, with 64.8% of patients achieving pCR with combination pembrolizumab and carboplatin/paclitaxel chemotherapy versus 51.2% in placebo arm^21^. Patient treatment selection directed by commercial PD-L1 biomarker assays with a > 1% immune cell staining cut point using the SP-142 Ventana assay are far from perfect, with only 8–20% of selected patients responding to treatment^22^. More significantly, the recent Impassion131 trial demonstrated no benefit of the addition of atezolizumab to paclitaxel in the PD-L1 + (or PD-L1 negative) population highlighting the need for improved biomarker driven patient selection^23^. Detailed spatial characterization of PD-L1 expressing cells within the tumour immune microenvironment (TIME) of TNBC may further our understanding of the important contribution of differing PD-L1 + cell phenotypes and their association with outcome. Multiplexed immunofluorescence (mIF) represents an advanced detection modality that allows for in-situ visualisation of multi-marker immunophenotypes within the TIME using unique spectrometry signatures^24^. Previous studies have provided proof of concept for routine clinical mIF, with no drop in performance as compared to current manual immunohistochemistry (IHC) techniques for TNBC samples^25–27^. The spatial information captured using mIF appears critical given evidence revealing its superior predictive value for PD-L1 responsiveness compared to standard monoplex PD-L1 IHC, tumour mutational burden or gene expression profiling in a meta-analysis of over 10 different solid tumours^27^. The aim of this study was to characterise the PD-L1 positive immune cell population and determine their prognostic significance in a retrospective cohort of Australian TNBC patients.

## Results

### TNBC patient cohort

The clinicopathological features of the patient cohort (n = 244) are as previously published^28^ and are summarised as follows (Supplementary Table S1 online): the average tumour size was 25.9 mm (range 7-120 mm), with patient age ranging from 25.9 to 96 years old. Median follow-up length was 4.3 years (range 0.02–16.3 years) for overall survival (OS, death from any cause) and breast cancer specific survival (BCSS, death directly attributable to breast cancer). 232 cases (95.1%) were grade 3, 85 cases (34.8%) had node-positive disease, and 111 (45.5%) had sTILs > 30%. 174 (71.3%) patients received adjuvant chemotherapy (regimens included cyclophosphamide, methotrexate 5-fluorouracil; anthracycline, cyclophosphamide; anthracycline, cyclophosphamide, paclitaxel; 5-fluorouracil, epirubicin, cyclophosphamide). Eighty six of 174 (49%) patients received a regimen which contained a taxane. Histologically, 221 (90.6%) were invasive ductal carcinoma of no special type with 17 (7%) metaplastic carcinomas and 6 (2.5%) other (apocrine, micropapillary, lobular). There were 71 deaths, 48 of which were breast cancer related. TNBC status was defined using current guidelines outlined by the College of American Pathologists (≤ 1% staining for ER and PR and HER2 negativity by IHC or silver in-situ hybridisation). All cases were scored for stromal TILs on the corresponding whole tumour section by an experienced breast Pathologist using standardised criteria^29^.

### Identification of specific stromal immune cell phenotypes

**Figure 1:**
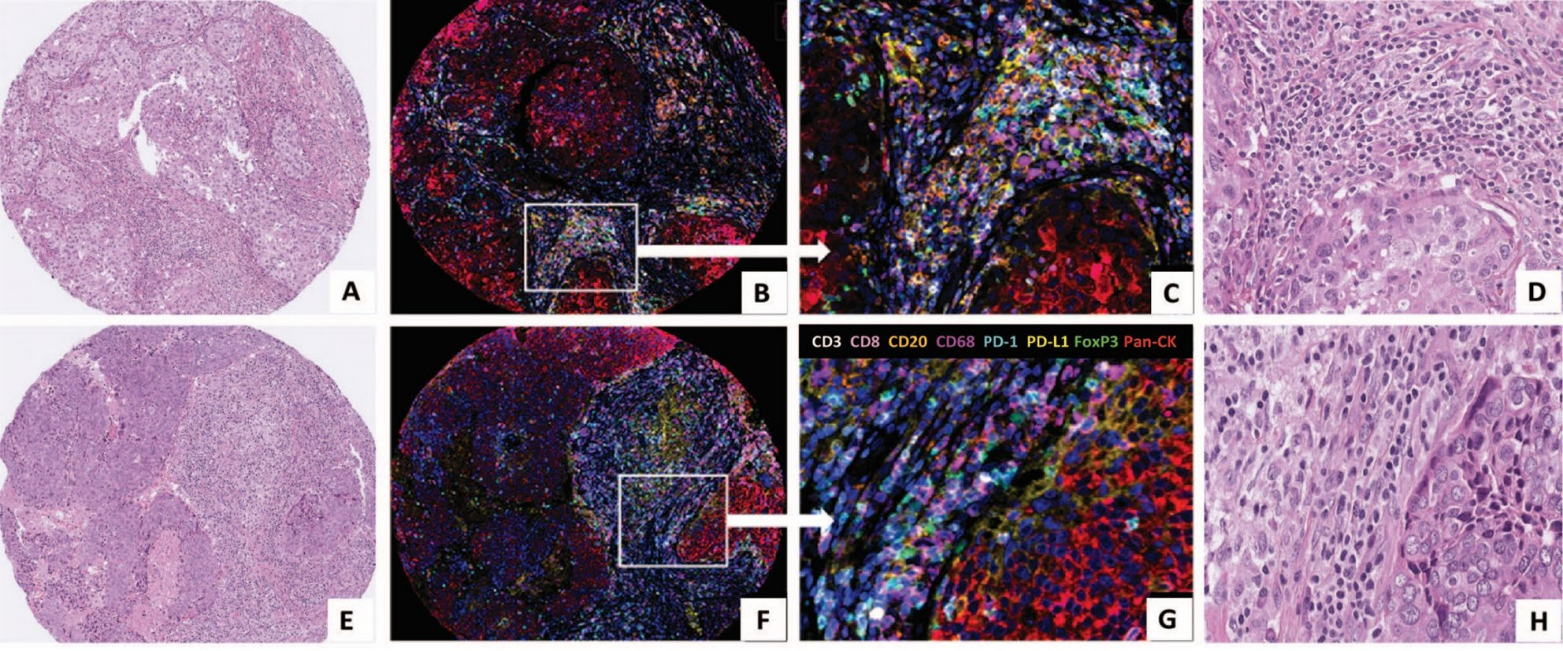
Matched haematoxylin & eosin (H&E) stained sections and multiplexed immunofluorescence (mIF) images from TMA cores of 2 patients tumours stained using Opal reagents: CD3 white, CD8 pink, CD20 orange, CD68 purple, PD-1 turquoise, PD-L1 yellow, FoxP3 green and Pan-CK red. DAPI nuclear counterstain is dark blue.. Patient 1 (**A**–**D**) & patient 2 (**E**–**H**) (**A**, **E**: H&E × 200; **B**, **F:** mIF × 200; **C** and **G:** mIF × 400; **D** and **H:** H&E × 400).

**Figure 2:**
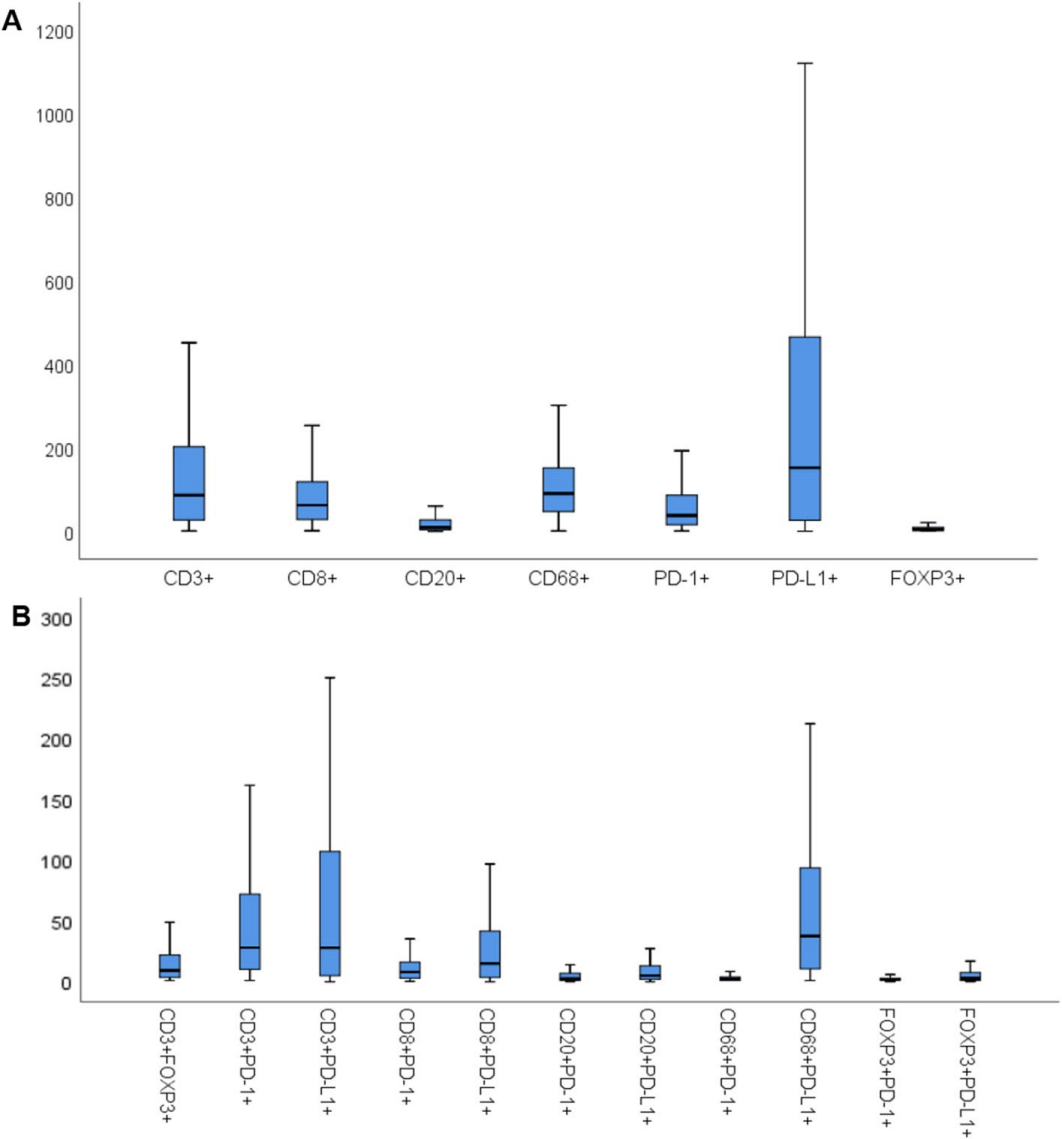
Box plots of key investigated stromal immune cell counts per TMA tissue core (cells/1.13 mm^2^) for single markers (**A**) and double markers (**B**). Outliers were excluded.

Using mIF, we were able to detect immunophenotypes defined by expression of up to four different markers predominantly within the stroma (Fig. 1). From the observed immunophenotypes, we investigated only those with the most clinical relevance, excluding inappropriate marker combinations (e.g., CD68^+^FOXP3^+^) and those with inadequate data points (less than one cell detected). The final immunophenotypes investigated and their median cell count densities (used as the cut-point value for subsequent analyses) are listed in Table 1, along with cell count distributions in Fig. 2. A bar chart summarising PD-L1 status according to immune marker phenotype in presented in Supplementary Fig. S1. Intra-epithelial cell counts for many samples were less than 1 and therefore not further assessed. Values from control normal breast tissue samples from a single patient are also included for comparison (Supplementary Table 4).

**Table 1:** Median stromal cell densities for key identified single, double, and triple marker immunophenotypes (cells/1.13 mm^2^) within the TNBC cohort.

Immunophenotype	TNBC stromal cell count
CD3^+^	86.67
CD8^+^	62.67
CD20^+^	9.33
CD68^+^	90.67
PD-1^+^	38.00
PD-L1^+^	152.33
FOXP3^+^	4.33
CD3^+^PD-1^+^	28.17
CD3^+^PD-L1^+^	28.00
CD3^+^FOXP3^+^	9.33
CD8^+^PD-1^+^	8.00
CD8^+^PD-L1^+^	15.00
CD20^+^PD-1^+^	2.58
CD20^+^PD-L1^+^	5.00
CD68^+^PD-1^+^	2.00
CD68^+^PD-L1^+^	37.67
FOXP3^+^PD-1^+^	2.83
FOXP3^+^PD-L1^+^	2.83
CD3^+^CD8^+^PD-1^+^	14.00
CD3^+^CD8^+^PD-L1^+^	16.00

Cell density measurements are for the stromal compartment. A PanCK^+^PD-L1^+^ median cell density of 80.00 cells/1.13 mm^2^ was detected in carcinoma epithelium.

### Correlations between immunophenotypes and clinicopathological features

Correlations between stromal immunophenotypes and clinicopathological data are presented in Supplementary Tables S2A-F online and are summarised as follows: the density of sTILs correlated positively with almost all single marker immunophenotypes, except in CD68^+^ and FOXP3^+^ cells. High PD-L1 expression was associated with chemotherapy administration (p = 0.020). High TILs consistently correlated with high densities of double marker T cell immunophenotypes, including CD3^+^PD-1^+^ (p < 0.001), CD3^+^PD-L1^+^ (p < 0.001), CD8^+^PD-1^+^ (p < 0.001), and CD8^+^PD-L1^+^ (p < 0.001). Notably, CD3^+^PD-1^+^ was also the only double marker immunophenotype positively correlated with chemotherapy administration (p = 0.011). PD-L1 co-localisation with CD68 was only associated with high TILs (p < 0.001), whilst with FOXP3, it was associated with smaller tumours (p = 0.028), negative nodal status (p = 0.009) and high sTILs (p = 0.017). CD3^+^CD8^+^PD-1^+^ (p < 0.001) and CD3^+^CD8^+^PD-L1^+^ (p < 0.001) only correlated with high sTILs.

### Correlations between CD68^+^*PD-L1*^+^ macrophages and other immunophenotypes

Given the significant results associated with CD68^+^PD-L1^+^ macrophages, we also investigated cell density correlations between CD68^+^PD-L1^+^ macrophages and other immunophenotypes (Supplementary Table S3A, B online). High CD68^+^PD-L1^+^ macrophages were associated with high CD3^+^ (p < 0.001), CD8^+^ (p = 0.001), PD-1^+^ (p < 0.001) and PD-L1^+^ (p < 0.001). Similar trends were observed amongst double marker immunophenotypes, with high CD68^+^PD-L1^+^ macrophages correlated with all T cell phenotypes, including CD3^+^PD-1^+^ (p < 0.001), CD3^+^PD-L1^+^ (p < 0.001), CD8^+^PD-1^+^ (p < 0.001) and CD8^+^PD-L1^+^ (p < 0.001). Positive associations were also observed with T_reg_ cells, including FOXP3^+^PD-L1^+^ (p < 0.001) and CD3^+^FOXP3^+^ (p < 0.001). Similarly, both CD3^+^CD8^+^PD-1^+^ (p < 0.001) and CD3^+^CD8^+^PD-L1^+^ (p < 0.001) were positively associated with CD68^+^PD-L1^+^.

### Prognostic significance of CD68^+^*PD-L1*^+^ macrophages amongst all TNBC patients

Cox regression survival models demonstrated that high stromal PD-L1 positivity was statistically significant for OS in univariate models as a single marker (Table 2; HR 0.60, 95% CI 0.36–0.96, p = 0.034), in addition to co-expression with CD68^+^ macrophages, i.e., a high CD68^+^PD-L1^+^ immunophenotype (Table 3; HR 0.59, 95% CI 0.36–0.96, p = 0.034). No other single marker immunophenotypes were significant in univariate models for OS or BCSS. Similarly, CD8^+^PD-L1^+^ was found to be non-significant in any univariate models for OS or BCSS (Table 3).

**Table 2:** Survival analysis of all single markers for overall survival and breast cancer-specific survival in all patients and in those who received chemotherapy.

	n	Overall survival			Breast cancer specific survival		
		HR	95% CI	p value	HR	95%CI	p value
**All patients**							
CD3^+^ (high vs. low)	121 vs. 120	0.84	0.53–1.35	0.479	0.96	0.54–1.71	0.881
CD8^+^ (high vs. low)	122 vs. 121	0.81	0.50–1.31	0.386	1.08	0.61–1.92	0.785
CD20^+^ (high vs. low)	121 vs. 118	0.96	0.60–1.54	0.852	1.12	0.63–2.00	0.708
CD68^+^ (high vs. low)	122 vs. 121	0.99	0.61–1.60	0.968	0.82	0.46–1.47	0.507
PD-1^+^ (high vs. low)	120 vs. 119	0.80	0.50–1.29	0.359	0.87	0.48–1.56	0.632
PD-L1^+^ (high vs. low)	122 vs. 121	0.60	0.36–0.96	**0.034**	0.60	0.33–1.09	0.093
FOXP3^+^ (high vs. low)	105 vs. 98	0.68	0.39–1.20	0.182	1.09	0.55–2.19	0.800
**Patients who received chemotherapy**							
CD3^+^ (high vs. low)	95 vs. 78	0.64	0.35–1.18	0.150	0.64	0.32–1.27	0.204
CD8^+^ (high vs. low)	97 vs. 77	0.61	0.33–1.13	0.115	0.70	0.35–1.39	0.312
CD20^+^ (high vs. low)	91 vs. 80	1.61	0.84–3.07	0.152	1.50	0.74–3.06	0.261
CD68^+^ (high vs. low)	87 vs. 87	0.90	0.48–1.69	0.741	0.80	0.40–1.59	0.522
PD-1^+^ (high vs. low)	88 vs. 83	0.63	0.34–1.18	0.152	0.72	0.36–1.44	0.346
PD-L1^+^ (high vs. low)	95 vs. 79	0.47	0.25–0.89	**0.020**	0.46	0.23–0.94	**0.033**
FOXP3^+^ (high vs. low)	78 vs. 67	0.58	0.26–1.26	0.170	0.81	0.34–1.97	0.645

**Table 3:** Survival models for CD68^+^PD-L1^+^ and CD8^+^PD-L1^+^ for all patients.

	n	Univariate analysis			MVA with CD68^+^PD-L1^+^			MVA with PD-L1^+^			MVA with CD8^+^PD-L1^+^		
		HR	95% CI	p value	HR	95% CI	p value	HR	95% CI	p value	HR	95% CI	p value
**Overall survival**													
Age (< 55 vs. ≥ 55)	105 vs. 138	0.48	0.29–0.80	**0.004**	0.46	0.26–0.78	**0.004**	0.40	0.23–0.67	**0.001**	0.45	0.22–0.93	**0.031**
Size (≤ 20 vs. > 20 mm)	113 vs. 129	0.54	0.32–0.89	**0.016**	0.53	0.31–0.92	**0.024**	0.54	0.32–0.93	**0.026**	0.54	0.29–1.04	0.066
LN status (neg vs. pos)	156 vs. 85	0.43	0.27–0.70	**0.001**	0.43	0.25–0.73	**0.002**	0.44	0.26–0.73	**0.002**	0.44	0.24–0.81	**0.008**
Chemotherapy (yes vs. no)	174 vs. 58	0.49	0.30–0.81	**0.006**							0.49	0.24–1.04	0.063
CD68^+^PD-L1^+^ (high vs. low)	116 vs. 115	0.59	0.36–0.96	**0.034**	0.56	0.33–0.95	**0.030**	–	–	–	–	–	–
PD-L1^+^ (high vs. low)	122 vs. 121	0.60	0.36–0.96	**0.034**	–	–	–	0.54	0.33–0.90	**0.018**	–	–	–
CD8^+^PD-L1^+^ (high vs. low)	104 vs. 103	0.82	0.47–1.41	0.467	–	–	–	–	–	–	1.07	0.57–1.99	0.838
**Breast cancer specific survival**													
Age (< 55 vs. ≥ 55)	105 vs. 138	0.50	0.27–0.91	**0.022**				0.41	0.21–0.77	**0.005**	0.55	0.25–1.23	0.147
Size (≤ 20 vs. > 20 mm)	113 vs. 129	0.45	0.24–0.85	**0.013**				0.51	0.26–0.99	**0.046**	0.54	0.25–1.17	0.119
LN status (neg vs. pos)	156 vs. 85	0.31	0.17–0.55	** < 0.001**	0.23	0.12–0.45	** < 0.001**	0.30	0.16–0.56	** < 0.001**	0.29	0.14–0.60	**0.001**
Chemotherapy (yes vs. no)	174 vs. 58	0.69	0.37–1.30	0.253	0.45	0.21–0.96	**0.039**				0.60	0.23–1.52	0.278
CD68^+^PD-L1^+^ (high vs. low)	116 vs. 115	0.58	0.31–1.06	0.075	0.47	0.25–0.88	**0.018**	–	–	–	–	–	–
PD-L1^+^ (high vs. low)	122 vs. 121	0.60	0.33–1.09	0.093	–	–	–	0.50	0.27–0.92	**0.026**	–	–	–
CD8^+^PD-L1^+^ (high vs. low)	104 vs. 103	1.01	0.52–1.95	0.975	–	–	–	–	–	–	1.12	0.53–2.33	0.770

LN, lymph node; MVA, multivariate analysis. Univariate and multivariate Cox regression models using a p value threshold of 0.05 for statistical significance, which are labelled in bold. Some multivariate models may not feature all significant p values, [–] indicates the immunophenotype was not significant.

**Figure 3:**
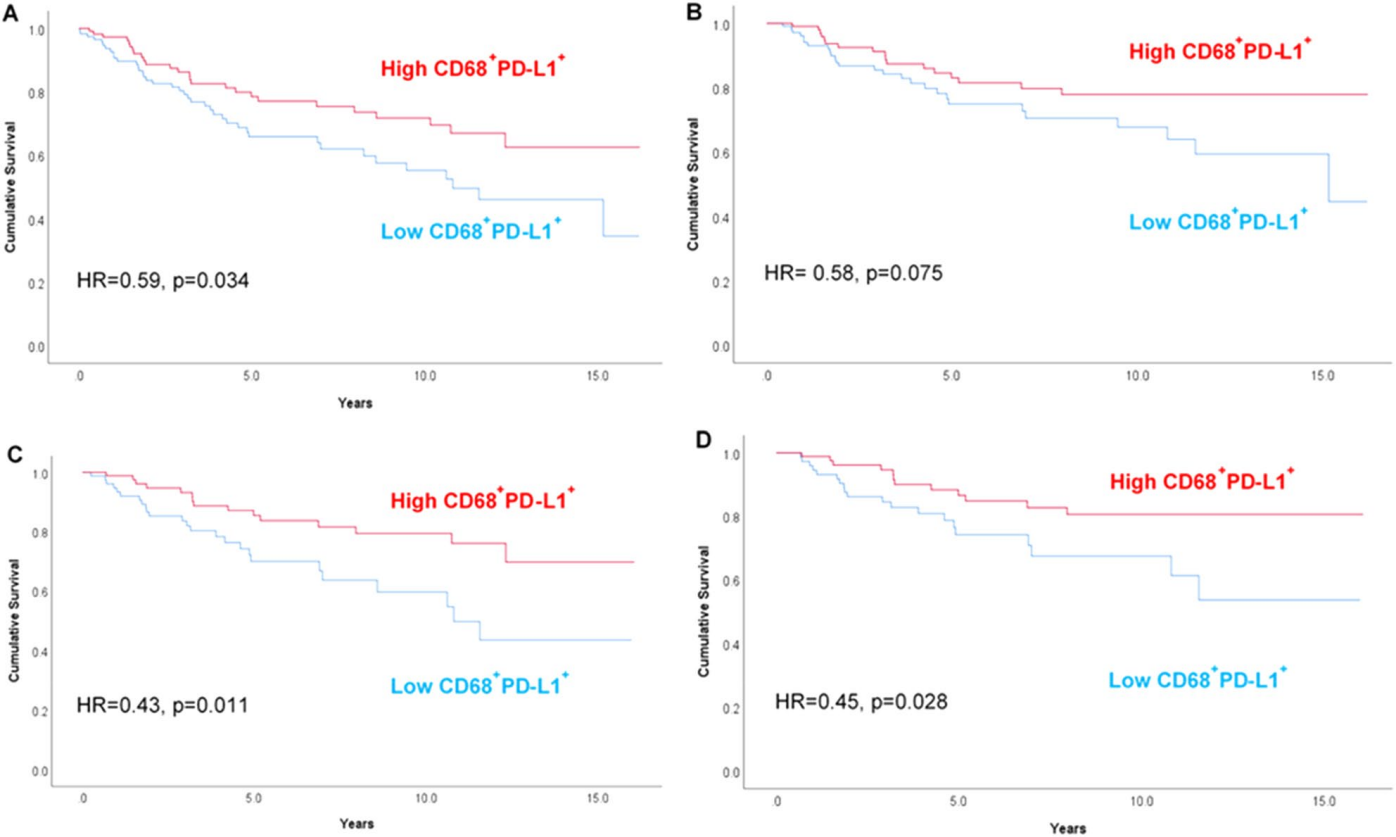
Kaplan–Meier plots stratified by stromal CD68^+^PD-L1^+^ for (**A**) overall survival and (**B**) breast cancer-specific survival in all patients; (**C**) overall survival and (**D**) breast cancer-specific survival for patients who received chemotherapy. A univariate Cox hazard ratio and p value is inserted within each graph.

Multivariate models identified that both high PD-L1^+^ (Table 3; HR 0.54, 95% CI 0.33–0.90, p = 0.018) and high CD68^+^PD-L1^+^ (Table 3 and Fig. 3A; HR 0.56, 95% CI 0.33–0.95, p = 0.030) phenotypes were independently predictive of OS in the whole patient cohort when controlled for clinicopathological features (age, size, LN status and chemotherapy administration). Likewise, both high PD-L1^+^ (HR 0.50, 95% CI 0.27–0.92, p = 0.026) and high CD68^+^PD-L1^+^ (HR 0.47, 95% CI 0.25–0.88, p = 0.018) phenotypes were also independently prognostic for improved outcome for BCSS (Fig. 3B), with CD68^+^PD-L1^+^ demonstrating incrementally improved prognostic value over PD-L1^+^ alone. CD8^+^PD-L1^+^ was not significant in any multivariate models. Kaplan–Meier curves for PD-L1 + alone are presented in Supplementary Fig. S2A, B.

### Prognostic value of CD68^+^*PD-L1*^+^ macrophages in patients receiving chemotherapy

Given the prognostic value of CD68^+^PD-L1^+^ macrophages, we further investigated this immunophenotype as a biomarker in patients who had received chemotherapy. Univariate models demonstrated that high PD-L1 expression was also associated with improved OS (Table 2; HR 0.47, 95% CI 0.25–0.89, p = 0.020), but the prognostic value was further improved with high CD68^+^PD-L1^+^ (Table 4; HR 0.43, 95% CI 0.23–0.82, p = 0.011). This same trend was observed in BCSS (PD-L1^+^ HR 0.46, 95% CI 0.23–0.94, p = 0.033; CD68^+^PD-L1^+^ HR 0.45, 95% CI 0.22–0.92, p = 0.028). No other single markers were found to be significant in univariate models. Administration of a taxane containing chemotherapeutic regime was not associated with outcome.

**Table 4:** Survival models for CD68^+^PD-L1^+^ and CD8^+^PD-L1^+^ in patients who received chemotherapy.

	n	Univariate analysis			MVA with CD68^+^PD-L1^+^			MVA with PD-L1^+^			MVA with CD8^+^PD-L1^+^		
		HR	95% CI	p value	HR	95% CI	p value	HR	95% CI	p value	HR	95% CI	p value
**Overall survival**													
Age (< 55 vs. ≥ 55)	93 vs. 81	0.55	0.30–1.03	0.060	0.45	0.23–0.88	**0.019**	0.39	0.20–0.76	**0.006**	0.31	0.14–0.68	**0.004**
Size (≤ 20 vs. > 20 mm)	83 vs. 90	0.42	0.21–0.82	**0.012**	0.47	0.23–0.98	**0.044**	0.43	0.21–0.90	**0.025**	0.41	0.18–0.93	**0.033**
LN status (neg vs. pos)	108 vs. 64	0.37	0.20–0.69	**0.002**	0.38	0.19–0.75	**0.006**	0.42	0.22–0.82	**0.011**	0.39	0.19–0.82	**0.013**
Taxane (yes vs. no)	86 vs. 88	1.16	0.60–2.20	0.65	–	–	–	–	–	–	–	–	–
CD68^+^PD-L1^+^ (high vs. low)	88 vs. 81	0.43	0.23–0.82	**0.011**	0.43	0.22–0.83	**0.012**	–	–	–	–	–	–
PD-L1^+^ (high vs. low)	95 vs. 79	0.47	0.25–0.89	**0.020**	–	–	–	0.45	0.23–0.86	**0.016**	–	–	–
CD8^+^PD-L1^+^ (high vs. low)	84 vs. 71	0.63	0.31–1.28	0.199	–	–	–	–	–	–	0.47	0.22–1.02	0.055
**Breast cancer specific survival**													
Age (< 55 vs. ≥ 55)	93 vs. 81	0.56	0.28–1.12	0.101				0.42	0.20–0.89	**0.023**	0.40	0.17–0.95	**0.038**
Size (≤ 20 vs. > 20 mm)	83 vs. 90	0.39	0.18–0.83	**0.015**				0.42	0.19–0.96	**0.041**	0.42	0.17–1.03	0.059
LN status (neg vs. pos)	108 vs. 64	0.29	0.14–0.59	**0.001**	0.27	0.13–0.56	** < 0.001**	0.33	0.12–0.70	**0.004**	0.30	0.13–0.71	**0.006**
Taxane (yes vs. no)	86 vs. 88	1.40	0.69–2.83	0.36	–	–	–	–	–	–	–	–	–
CD68^+^PD-L1^+^ (high vs. low)	88 vs. 81	0.45	0.22–0.92	**0.028**	0.38	0.18–0.78	**0.009**	–	–	–	–	–	–
PD-L1^+^ (high vs. low)	95 vs. 79	0.46	0.23–0.94	**0.033**	–	–	–	0.42	0.20–0.87	**0.020**	–	–	–
CD8^+^PD-L1^+^ (high vs. low)	84 vs. 71	0.77	0.36–1.67	0.510	–	–	–	–	–	–	0.61	0.26–1.41	0.245

LN, lymph node; MVA, multivariate analysis. Univariate and multivariate Cox regression models using a p value threshold of 0.05 for statistical significance, which are labelled in bold; [–] indicates the immunophenotype was not significant.

In multivariate models (Table 4), there was a clear OS benefit in patients expressing high numbers of PD-L1^+^ (HR 0.45, 95% CI 0.23–0.86, p = 0.016) and CD68^+^PD-L1^+^ cells (HR 0.43, 95% CI 0.22–0.83, p = 0.012). Likewise, both PD-L1^+^ (HR 0.42, 95% CI 0.20–0.87, p = 0.020) and CD68^+^PD-L1^+^ (HR 0.38, 95% CI 0.18–0.78, p = 0.009) were independently prognostic for BCSS, with the predictive power of CD68^+^PD-L1^+^ incrementally improved over PD-L1 alone. CD8^+^PD-L1^+^ was not significant for patients who received chemotherapy. The OS and BCSS survival benefit of high CD68^+^PD-L 1^+^ macrophage counts is illustrated in Fig. 3C, D. Kaplan–Meier curves for PD-L1 + alone are presented in Supplementary Fig. S2C, D.

## Discussion

Despite its overall poor survival, a sub-group of TNBC patients have a good prognosis and respond well to standard of care chemotherapy, often correlating with high stromal TILs levels. Current guidelines for TILs assessment however do not use immunophenotypic data to assess prognostic significance and no other biomarkers are currently in routine clinical use to help guide treatment planning for these patients. Combined chemotherapy and immunotherapy for advanced and early-stage TNBC has shown a survival benefit for patients with positive PD-L1 expression (e.g., SP142 immune cell > 1%), although response rates in the positive patient group vary widely. Combined assessment of PD-L1 status and TILs density in TNBC has therefore been recommended to help improve patient selection for immunotherapy^30,31^ but problems in PD-L1 clinical assays remain^32^. Our study utilises a cell median cut-point definition of PD-L1 positivity and not > 1% immune cell staining, as used in commercial PD-L1 assays such as SP142. Each of the commercially assays (SP142, SP263 both Ventana, 22C3 and 28–8 both Dako) shows variability in detection threshold and variable agreement between clones, which differs significantly from the methods used in the current study, limiting comparisons with studies using these methods^33^. Additionally the epitope target of PD-L1 varies between antibodies, although there is some overlap between the antibody used in our study (E1L3N) and SP142 and SP263 which bind to non-identical epitopes in the cytoplasmic C-terminus domain (22C3 and 28–8 bind to the extracellular domain).^34^ Improved characterisation of the stromal PD-L1 immunophenotypes present in TNBC may highlight the importance of the cellular context of PD-L1 expression and potentially also provide data to support the use of immune markers as new routine biomarkers for all patients with TNBC, irrespective of the indications for immunotherapy. PD-L1 (CD274) is a cell surface molecule of the B7 family that may be expressed by immune cells and cancer epithelial cells to inhibit T cell proliferation and induce apoptosis upon binding to its ligand PD-1^35^. Using a retrospective cohort of 244 patients with TNBC, our data demonstrates that high stromal CD68^+^PD-L1^+^ macrophages have incrementally improved prognostic significance to that provided by PD-L1 stromal expression alone or in any other key cellular context, e.g., stromal CD8^+^PD-L1^+^ T cells, carcinoma epithelial PD-L1^+^. High PD-L1^+^ is usually associated with high TILs and a more favourable prognosis^36–38^, with a high CD8^+^ and PD-L1^+^ population (using monoplex IHC) shown to be prognostically beneficial in an analysis of the IMpassion130 cohort (HR 0.64, 95% CI 0.49–0.83)^39^. However, our data does not find a significant association of outcome with CD8^+^PD-L1^+^ expression and suggests that CD68^+^ macrophages carry prognostic significance. Prior studies have also identified a role of high CD8 + CD103 + resident memory T-cells (Trm) to be an important predictor of improved outcome in TNBC, but we were unable to address this cellular phenotype in our study due limitations in the number of available targets that could be assessed^40^. The role of macrophages in TNBC was highlighted in a recent single-arm study of 45 TNBC patients receiving neoadjuvant durvalumab and nab-paclitaxel, with high CD68^+^PD-L1^+^ (both stromal and epithelial) associated with improved rates of pCR (73.33% vs. 23.33%, p = 0.053)^41^. Similar findings from the same group have highlighted the improved prognosis which CD68^+^PD-L1^+^ cells carry in non-small cell lung cancer when treated with immunotherapy^42^. Additionally, one other retrospective study of 76 patients in all breast cancer subtypes (23 TNBC cases) found improved rates of pCR with neoadjuvant chemotherapy (74.3% vs. 40%) were associated with high CD68 and PD-L1 expression using monoplex IHC^43^.

Macrophages are typically subclassed within the TIME as classically activated, anti-tumour M1- or alternatively activated, pro-tumour M2-like macrophages, also known as tumour associated macrophages (TAMs). Higher proportions of CD68^+^ macrophages are seen in TNBC compared to hormone-positive disease, with higher reported unpolarised M0- and M1-like subtype proportions^44–46^. However, further studies have shown that TNBC may selectively cause unpolarised macrophages to become TAMs, and thus other studies have shown TAMs to be in greater proportions^47,48^.

The effects of the M2-like phenotype have been more closely linked to cancer progression and metastasis and therefore patient prognosis with *SPINK1*, *LAMC2*, *IGFBP1,* and *IL-23A* gene expression^47^. A seminal study by Leek et al. discovered associations between increased macrophage density and angiogenesis, leading to poorer survival outcomes^49^. This implies that macrophages play a pro-tumour role by upregulating endothelial genes, which were later identified as *ECSCR, ANGPTL4* and *ITGB4*^47^. Upregulation of PD-1 and PD-L1 expression is also seen with the presence of TAMs^50^. In the TIME, anti-tumour M1 macrophages promote a cytotoxic response mediated by classic inflammatory cytokines tumour necrosis factor- ⍺ (TNF-⍺), interleukin (IL) -1, IL-12, and IL-23^51^. Contrastingly, pro-tumour TAMs recruit more T_reg_ cells and induce apoptosis in CTLs through IL-4 and IL-10^52,53^.

CD163 is a haemoglobin scavenger macrophage receptor that binds to haptoglobin-haemoglobin complexes, and has been used to selectively study M2-like macrophages in TNBC^54^. Recent studies have concluded that high levels of CD163^+^ TAMs were both associated with poorer rates of pCR in patients receiving neoadjuvant anthracycline- and taxane-based chemotherapy, as well as shorter OS and relapse-free survival (RFS)^55–57^. Furthermore, high CD163^+^ TAMs combined with a low CD4^+^, CD8^+^, and CD20^+^ TILs signature was also associated with poorer OS and RFS^58^. These results imply the direction of association is reversed in the M2 macrophage subclass and therefore further study is warranted to substantiate these findings in larger cohorts. CD204, a Class A scavenger receptor associated with angiogenesis, immunosuppression and further tumour proliferation, is another TAM marker used in studies investigating invasive breast cancer, with results again suggesting that high macrophage counts were also associated with poorer prognosis^59–61^. However, none have looked specifically at TNBC. The timing of chemotherapy (i.e. adjuvant or neoadjuvant) seems to have no effect on the prognostic significance, increasing the value macrophages may have as a clinical biomarker. Single cell RNA-sequencing has found smaller proportions of TAMs within the TIME of TNBC, however high expression of the M2 subtype is associated with poorer overall survival (p = 0.002)^46^. An analysis of metastatic TNBC found non-response to neoadjuvant nab-paclitaxel and pembrolizumab resulted in a lower CD68 signature with higher *CSF1R*- expressing TAMs^62^. In contrast, treatment response was associated with M1 subtype prevalence expressing *CXCL9, CXCL10, and HLA-DR*.

Given the critical role macrophages have in the TIME, two studies have investigated their effectiveness as targets of immune checkpoint inhibition in TNBC. Preliminary results from a phase II trial have shown that lacnotuzumab (MCS110) in combination with carboplatin/gemcitabine currently provides little benefit for patients with advanced TNBC whilst cabiralizumab will be combined with nivolumab and neoadjuvant carboplatin/paclitaxel for stage II or III TNBC in a trial that is still currently recruiting^63,64^. Both drugs target colony stimulating factor 1 (CSF1). The rationale behind TAM-targeted immunotherapy has six potential mechanisms: suppressing macrophage recruitment, accelerating macrophage apoptosis, inhibiting pro-tumour activities, repolarisation back to M1-like phenotypes, aiding cancer cell phagocytosis, and chimeric antigen receptor macrophage (CAR-M) development^65^. It is yet to be seen which one of these mechanisms will be most effective in improving patient clinical outcomes.

The adoption of mIF and digital pathology brings unique benefits and challenges to solid tumour analysis. Biomarker quantitation using artificial intelligence-driven image analysis is now a reality and will develop into clinical algorithms once standardised and appropriately validated in the near future. The Vectra Polaris™ platform used in this study allows for up to eight targets to be simultaneously visualised on a single specimen, facilitating quantitative spatial analysis and immunophenotype identification^66,67^. It is the foremost used mIF system and has been successfully applied to various solid tumours for research purposes^25,26,66,68,69^. Newer systems, such as Akoya CODEX™, can accommodate up to 40 markers and studies have begun to use this system^67,70^. However, greater standardisation and automation of staining and imaging protocols will further validate the clinical applicability of mIF, including the choice of assay. The potential overlapping of wavelengths associated with each fluorophore can also interfere with spectra detection, with cell detection potentially being problematic and incorrect cell classifications being made. Farkas et al. found a panel utilising greater than seven markers could be problematic and therefore a need for studies of carefully chosen immunofluorescence targets will be required before clinical adoption can occur to avoid crossover and optimise image analysis^24,71^. The selection of regions of interest (ROI) to be analysed is also important to analyse a truly representative sample of the tumour, avoiding hotspots or areas devoid of any immune cell activity. This is one short coming of our study, which uses TMA cores and not whole section images. The variability in immune cell density amongst selected ROIs may be greater, depending on tumour heterogeneity. A recent study comparing mIF to H&E stromal TILs and the SP142 PD-L1 IHC assay found 15 high power fields of the tumour were required to optimise accuracy^72^. Additionally, spatial relationships such as the distance between neighbouring immune cells and the tumour interface, will likely yield further prognostic information but the vast quantities of data will require interrogation using advanced deep learning artificial intelligence algorithms to define their clinical significance^73^. Whilst such algorithms will likely develop and reach clinical application as the uptake of digital pathology gathers pace, until then simplified panels of immune cells will continue to be used for clinical reporting by Pathologists. Our data provides evidence to suggest that a multiplexed panel of CD68 and PD-L1 could potentially improve upon monoplex PD-L1 assays as a general prognostic marker in TNBC. Studies involving cohorts from immunotherapy trials will be required to determine if this may also provide improved predictive value over PD-L1 expression alone to guide patient selection for immune checkpoint therapy.

Differences in methodology in PD-L1 cut-point assessment between studies from clinical trials (e.g. > 1% immune cell staining) and those using cell count /density measurement also limit the comparisons which can be made between such studies.

## Conclusion

High dual stained stromal CD68^+^PD-L1^+^ macrophages identifies a subgroup of TNBC patients associated with improved prognosis and incrementally improved predictive value over PD-L1^+^ alone for BCSS, in multivariate models accounting for age, size, LN status and chemotherapy status. This prognostic immunophenotype provides useful data to further stratify TNBC outcome and aid in decision making for patients under consideration for standard of care chemotherapy.

## Methods

### Tissue microarray construction

Tissue microarrays (TMAs) were constructed using a Beecher Manual Arrayer MTA-1 (Beecher Instruments, Inc., Sun Prairie, WI, USA), with appropriate areas sampled from the periphery of the tumour block marked up by a breast Pathologist on a H&E slide. Furthermore, 3 cores of normal spleen, 1 core of normal kidney, and normal breast tissue from 1 patient, were included in each of the 9 TMAs as controls. Paraffin sections were cut at 4 μm onto Superfrost™ glass slides (ThermoFisher Scientific, Waltham, MA, USA) and stained for H&E using a Leica automated staining machine (Leica Biosystems, Wetzlar, Germany) in the Department of Anatomical Pathology, NSW Health Pathology, St George Hospital, Kogarah, Australia.

### Multiplexed immunofluorescence

Staining of tissue was performed on slides that were baked in the oven at 58 °C for 60 min and rehydrated in Gemini AS Automated Stainer (Epredia, Kalamazoo, MI, United States of America), using the following steps: Xylene 2 × 5 min, 100% Ethanol 3 × 1 min, 70% Ethanol 1 × 1 min and distilled water 1 × 1 min followed by 10 min wash in distilled water.

The staining conditions for all antibodies were first tested using chromogenic 3,3′‐ diaminobenzidene (DAB) detection (BOND Polymer Refine detection, Leica Biosystems. #DS9800). Initial antigen retrieving step was performed in Decloaking Chamber™ NxGen (Biocare Medical, Pancheco, CA, United States of America) in citrate followed by EDTA based antigen retrieving buffers (DAKO, AR6 #K8005 and AR9 #K8004) in 110 °C for 5 min. Staining was completed on Leica Bond RX automated immunostainer (Leica Biosystems, Australia). Triple negative breast cancer tissue was used for all optimisation steps. Localisation of IHC staining signal and quality was used as a baseline for comparison for mIF staining.

The TSA‐based Opal 9 multiplexing technology was used for immunofluorescence staining (Opal 7‐Color Automation IHC Kit, # NEL821001KT; Opal Polaris 480 reagent pack, # FP1500001KT and Opal Polaris 780 reagent pack # FP1501001KT: Akoya Biosciences, Marlborough, MA, USA). Primary antibody conditions determined in the initial DAB optimisation step were applied to the Opal monoplex and multiplex optimisation.

Each biomarker antibody was paired with an individual Opal fluorophore (Table 5). Pairing of antibody-Opal fluorophore was based on the biomarker co-expression in the tissue and their expected levels of protein expression. Biomarkers expressed in the same compartment were paired with spectrally distanced Opal fluorophores. The Opal fluorophores were used in 1:150 dilution except for Opal Polaris 780 with TSA-DIG 1:150 and Opal Polaris 780 1:25. DAPI was used as a nuclear counterstain. All staining was performed on a Leica Bond RX autostainer (Leica Biosystems, Australia).

**Table 5:** Multiplexed immunofluorescence antibodies and their targets.

Target	Antibody clone	Source	Dilution and antigen retrieval	Opal fluorophores
CD3	F7.2.38	DAKO M7254	1:100, E-AR	Opal 780
CD8	C8/144B	Invitrogen MA5-13473	1:100, E-AR	Opal 690
CD20	EP459Y	Abcam ab78237	1:200, C-AR	Opal 620
CD68	CD68/684	Abcam ab201340	1:4000, C-AR	Opal 480
PD-1	EPR4877(2)	Abcam ab137132	1:50, C-AR	Opal 520
PD-L1	E1L3N	Cell signalling #13684	1:25, E-AR	Opal 570
FOXP3	236A/E7	Abcam ab20034	1:1000, E-AR	Opal 540
Pan-CK	AE1/AE3	Abcam ab27988	1:3000, C-AR	Opal 650

C-AR, citrate antigen retrieval at pH 6.0; E-AR, Ethylenediaminetetraacetic acid (EDTA) antigen retrieval at pH 9.0; Pan-CK, pan-cytokeratin; PD-1, programmed cell death protein-1; PD-L1, programmed death-ligand 1.

In the process of achieving Opal monoplex optimisation, each biomarker was assessed for staining quality and intensity. Acquired monoplex images were unmixed and analysed with Akoya’s INFORM software version 2.5.1. Due to the Opal signal and antibody concentration variability we aimed to get an optimal normalised count range of 10–20 while signal to noise ratio assessed by measuring the positive signal versus background with ratio of 10:1 deemed as sufficient. On completion of satisfactory monoplex IF, the mIF protocol was performed using all 8 antibodies on each slide of the TMA cohort. Cores of normal spleen and normal breast tissue were used as internal controls in the TMA slides to ensure staining intensity was comparable across all slides. Antibody concentrations were further adjusted in the multiplex round in normalised counts not meeting the criteria.

### Image analysis

Fluorescent slides were scanned using the Vectra Polaris 3.0 (Akoya Biosciences, Marlborough, MA, United States of America) using 40 × magnification (Plan APO 40 × /NA 0.75, 0.25um/pixel) and auto-estimated exposure times. Whole slide scan was imaged using 5 epi-fluorescent filters (DAPI, Opal 480, Cy3, Cy5 and Opal 780). Individual TMA cores were selected using the TMA array in the Phenochart software for image acquisition and acquired with auto-estimated exposure times for each epi-fluorescent filter. The full Opal 9 acquisition protocol requires use of 7 epi-fluorescent filters (DAPI, Opal 480, FITC, Cy3, Texas Red, Cy5 and Opal 780) imaging at 20 nm spectral bands as designed for the Vectra Polaris. Multiplex auto-fluorescent slide with no primary antibodies was created and scanned using the same exposure times as labelled multiplex slides. Previously created and assessed spectral library for Opal 9 panel and the auto-fluorescent slide were used for unmixing of the MSI core images in INFORM software. Images of individual and combined colour channels for CD8, CD68 and PD-L1 are presented in Supplementary Fig. S3A–E.

Individual TMA images derived from mIF staining were analysed using an open-source digital image analysis software platform QuPath v0.2.3 (https://qupath.github.io/)74. Tissue detection and segmentation into stroma and tumour epithelium was created using trainable machine learning algorithms in the pixel classifiers. Pan-cytokeratin staining was used to guide tumour epithelium and stroma segmentation, supervised by a Pathologist. Cell segmentation was based on DAPI nuclear staining using the inbuild cell detection algorithm. Two different cell detection algorithms were derived, one for tumour and one for stroma. Phenotyping of all biomarkers was created using the latest multiplex analysis approach available in QuPath v0.2.3, by creating object classifiers. For object classification, we utilised the machine learning algorithms available (random forest). Each classifier was thoroughly trained and verified on multiple selected cell measurements. The combined classifier was applied to each TMA core.

Cell counts for all targets were provided for stromal and epithelial compartments per TMA core (average core diameter 1.2 mm = 1.13 mm^2^). The median cell count value was used as the cut-point for all analyses.

### Statistics

Preliminary associations between specific immunophenotype combinations and clinicopathological features were first investigated with a Chi-squared test. Univariate and multivariate OS and BCSS analyses were conducted using Cox proportional hazards modelling, with a p < 0.05 considered significant. A backward selection method was applied to find the most appropriate multivariate models by elimination of redundant variables. Survival predictions were represented with Kaplan–Meier plots. Statistical analysis was completed using IBM SPSS Statistics 26 (IBM Corp., Armonk, NY, USA).

### Ethics

Ethics approval was granted by the South Eastern Sydney Local Health District Human Research Ethics Committee at the Prince of Wales Hospital, Sydney (Boost: HREC 96/16 and TNBC: HREC 2018/ETH00138) who granted a waiver of consent to perform research analyses on the tissue blocks. All methods were performed in accordance with the relevant institutional guidelines and regulations.

## Supplementary Information

Supplementary Information.

## Data Availability

The data is not publicly available due to ethics restrictions but may be accessible on reasonable request to the corresponding author.

## Abbreviations

BCSS: Breast cancer-specific survival
CI: Confidence interval
DAB: Diaminobenzidine
ER: Oestrogen receptor
FFPE: Formalin fixed paraffin embedded
H&E: Haematoxylin & eosin
HER2: Human epidermal growth factor receptor 2
HR: Hazard ratio
IHC: Immunohistochemistry
LN: Lymph node
mIF: Multiplexed immunofluorescence
OS: Overall survival
pCR: Pathological complete response
PD-1: Programmed cell death protein 1
PD-L1: Programmed death-ligand 1
PR: Progesterone receptor
RFS: Relapse free survival
ROI: Region of interest
sTILs: Stromal tumour infiltrating lymphocytes
TAMs: Tumour associated macrophages
TILs: Tumour infiltrating lymphocytes
TIME: Tumour immune microenvironment
TMA: Tissue microarray
TNBC: Triple negative breast cancer
T_reg_: Regulatory T-cell
