# Supplementary material for: Multiplexed immunofluorescence identifies high stromal CD68+PD-L1+ macrophages as a predictor of improved survival in triple negative breast cancer
Source: Sci Rep. 2021 Nov 3;11:21608. doi: 10.1038/s41598-021-01116-6 (PMC8566595; doi:10.1038/s41598-021-01116-6)
Supplement: Supplementary file 1 — Supplementary Information. [file 41598_2021_1116_MOESM1_ESM.docx]

**Multiplexed immunofluorescence identifies high stromal**

**CD68^+^PD-L1^+^ macrophages as a predictor of improved survival in triple negative breast cancer**

**James Wang^1^, Lois Browne^2^, Iveta Slapetova^3^, Fei Shang^3^, Kirsty Lee^4^, Jodi Lynch^1,2^, Julia Beretov^1,2,5^, Renee Whan^3^, Peter Graham^1,2^, Ewan KA Millar^1,5-7*^**

**Supplementary Table S1.** Clinicopathological features of the TNBC patient cohort.

|  | **Number** | **Percentage (%)** |
| --- | --- | --- |
| **Age** |  |  |
| <55 | 105 | 43.0 |
| ≥55 | 138 | 57 |
| **Grade** |  |  |
| 2 | 12 | 5 |
| 3 | 232 | 95 |
| **Histology** |  |  |
| IDC-NST | 221 | 91 |
| Metaplastic | 17 | 7 |
| Other | 6 | 2 |
| **Size** |  |  |
| ≤20 | 113 | 47 |
| >20 | 129 | 53 |
| **Lymph Node Status** |  |  |
| Negative | 156 | 65 |
| Positive | 85 | 35 |
| **Chemotherapy** |  |  |
| Received | 174 | 75 |
| Not Received | 58 | 25 |
| **TILs** |  |  |
| < 30% | 133 | 55 |
| ≥ 30% | 111 | 45 |
| **Deaths** |  |  |
| Yes | 71 | 29 |
| No | 173 | 71 |

IDC-NST, Invasive ductal carcinoma of no special type. Some variables have a small number of missing data points.

**Supplementary Table S2A.** **X^2^** analysis for stromal single marker phenotypes.

|  | **CD3^+^** | | | **CD8^+^** | | | **CD20^+^** | | | **CD68^+^** | | |
| --- | --- | --- | --- | --- | --- | --- | --- | --- | --- | --- | --- | --- |
|  | **High** | **Low** | **X^2^, p value** | **High** | **Low** | **X^2^, p value** | **High** | **Low** | **X^2^, p value** | **High** | **Low** | **X^2^, p value** |
| **Age** |  |  | 0.17, 0.683 |  |  | 0.29, 0.592 |  |  | 1.80, 0.180 |  |  | <0.01, 0.986 |
| < 55 | 54 | 50 |  | 55 | 50 |  | 58 | 46 |  | 53 | 52 |  |
| ≥ 55 | 67 | 69 |  | 67 | 70 |  | 63 | 71 |  | 69 | 68 |  |
| **Size** |  |  | 0.51, 0.473 |  |  | 0.04, 0.850 |  |  | 0.36, 0.551 |  |  | 0.96, 0.327 |
| ≤ 20mm | 59 | 53 |  | 57 | 56 |  | 59 | 53 |  | 61 | 52 |  |
| > 20mm | 61 | 66 |  | 63 | 65 |  | 61 | 64 |  | 61 | 67 |  |
| **LN status** |  |  | 4.30, 0.038 |  |  | 0.10, 0.757 |  |  | 0.98, 0.332 |  |  | 0.16, 0.686 |
| Negative | 70 | 84 |  | 77 | 78 |  | 73 | 79 |  | 76 | 79 |  |
| Positive | 50 | 34 |  | 44 | 41 |  | 46 | 38 |  | 44 | 41 |  |
| **Chemotherapy** |  |  | 5.13, 0.023 |  |  | 6.14, 0.013 |  |  | 1.24, 0.265 |  |  | 0.01, 0.908 |
| Yes | 95 | 78 |  | 97 | 77 |  | 91 | 80 |  | 87 | 87 |  |
| No | 21 | 35 |  | 21 | 36 |  | 25 | 31 |  | 29 | 28 |  |
| **TILs** |  |  | 42.66, <0.001 |  |  | 22.15, <0.001 |  |  | 5.24, 0.022 |  |  | 3.51, 0.061 |
| < 30% | 40 | 90 |  | 48 | 84 |  | 57 | 73 |  | 59 | 73 |  |
| ≥ 30% | 81 | 30 |  | 74 | 37 |  | 64 | 45 |  | 63 | 48 |  |
| **Histology** |  |  | 4.31, 0.038 |  |  | 1.85, 0.173 |  |  | 4.48, 0.034 |  |  | 9.92, 0.002 |
| IDC | 115 | 105 |  | 114 | 107 |  | 115 | 103 |  | 118 | 103 |  |
| Other | 6 | 15 |  | 8 | 14 |  | 6 | 15 |  | 4 | 18 |  |

**Supplementary Table S2B.** **X^2^** analysis for stromal single marker phenotypes.

|  | **PD-1^+^** | | | **PD-L1^+^** | | | **FOXP3^+^** | | |
| --- | --- | --- | --- | --- | --- | --- | --- | --- | --- |
|  | **High** | **Low** | **X^2^, p value** | **High** | **Low** | **X^2^, p value** | **High** | **Low** | **X^2^, p value** |
| **Age** |  |  | 1.06, 0.303 |  |  | <0.01, 0.986 |  |  | 0.58, 0.447 |
| < 55 | 48 | 55 |  | 53 | 52 |  | 41 | 43 |  |
| ≥ 55 | 72 | 63 |  | 69 | 68 |  | 64 | 54 |  |
| **Size** |  |  | 0.01, 0.937 |  |  | 0.04, 0.850 |  |  | 0.72, 0.395 |
| ≤ 20mm | 56 | 54 |  | 56 | 57 |  | 55 | 45 |  |
| > 20mm | 64 | 63 |  | 65 | 63 |  | 50 | 52 |  |
| **LN status** |  |  | 1.94, 0.163 |  |  | 0.25, 0.617 |  |  | 0.30, 0.586 |
| Negative | 71 | 80 |  | 80 | 75 |  | 72 | 63 |  |
| Positive | 48 | 37 |  | 41 | 44 |  | 32 | 33 |  |
| **Chemotherapy** |  |  | 0.18, 0.673 |  |  | 5.41, 0.020 |  |  | 0.21, 0.648 |
| Yes | 88 | 83 |  | 95 | 79 |  | 78 | 67 |  |
| No | 27 | 29 |  | 21 | 36 |  | 24 | 24 |  |
| **TILs** |  |  | 57.63, <0.001 |  |  | 73.44, <0.001 |  |  | 2.65, 0.104 |
| < 30% | 35 | 93 |  | 33 | 99 |  | 48 | 56 |  |
| ≥ 30% | 85 | 26 |  | 89 | 22 |  | 57 | 42 |  |
| **Histology** |  |  | 7.32, 0.007 |  |  | 3.27, 0.070 |  |  | 0.17, 0.678 |
| IDC | 115 | 102 |  | 115 | 106 |  | 99 | 91 |  |
| Other | 5 | 17 |  | 7 | 15 |  | 6 | 7 |  |

**Supplementary Table S2C.** **X^2^** analysis for stromal double marker phenotypes.

|  | **FOXP3^+^PD-L1^+^** | | | **CD68^+^PD-L1^+^** | | | **CD20^+^PD-1^+^** | | | **CD20^+^PD-L1^+^** | | |
| --- | --- | --- | --- | --- | --- | --- | --- | --- | --- | --- | --- | --- |
|  | **High** | **Low** | **X^2^, p value** | **High** | **Low** | **X^2^, p value** | **High** | **Low** | **X^2^, p value** | **High** | **Low** | **X^2^, p value** |
| **Age** |  |  | 1.04, 0.307 |  |  | 0.01, 0.908 |  |  | 4.67, 0.031 |  |  | 5.86, 0.016 |
| < 55 | 34 | 28 |  | 50 | 50 |  | 30 | 19 |  | 43 | 24 |  |
| ≥ 55 | 36 | 42 |  | 66 | 64 |  | 22 | 33 |  | 30 | 39 |  |
| **Size** |  |  | 4.83, 0.028 |  |  | 0.01, 0.938 |  |  | 0.35, 0.556 |  |  | 0.05, 0.824 |
| ≤ 20mm | 42 | 29 |  | 55 | 53 |  | 27 | 24 |  | 35 | 29 |  |
| > 20mm | 28 | 41 |  | 61 | 60 |  | 25 | 28 |  | 38 | 34 |  |
| **LN status** |  |  | 6.76, 0.009 |  |  | 0.76, 0.384 |  |  | 3.63, 0.057 |  |  | 2.08, 0.149 |
| Negative | 52 | 38 |  | 71 | 76 |  | 27 | 37 |  | 40 | 42 |  |
| Positive | 17 | 32 |  | 44 | 37 |  | 24 | 15 |  | 32 | 20 |  |
| **Chemotherapy** |  |  | <0.01, 1.000 |  |  | 0.57, 0.451 |  |  | 0.55, 0.460 |  |  | 0.55, 0.457 |
| Yes | 53 | 53 |  | 88 | 81 |  | 40 | 37 |  | 59 | 50 |  |
| No | 14 | 14 |  | 23 | 27 |  | 9 | 12 |  | 10 | 12 |  |
| **TILs** |  |  | 5.72, 0.017 |  |  | 51.55, <0.01 |  |  | 0.65, 0.420 |  |  | 1.33, 0.249 |
| < 30% | 23 | 37 |  | 33 | 87 |  | 18 | 22 |  | 21 | 24 |  |
| ≥ 30% | 47 | 33 |  | 83 | 28 |  | 34 | 30 |  | 52 | 39 |  |
| **Histology** |  |  | <0.01, 1.000 |  |  | 2.23, 0.136 |  |  | 1.89, 0.169 |  |  | 0.05, 0.830 |
| IDC | 66 | 66 |  | 110 | 103 |  | 51 | 48 |  | 69 | 59 |  |
| Other | 4 | 4 |  | 6 | 12 |  | 1 | 4 |  | 4 | 4 |  |

**Supplementary Table S2D.** **X^2^** analysis for stromal double marker phenotypes.

|  | **CD3^+^FOXP3^+^** | | | **CD8^+^PD-1^+^** | | | **CD8^+^PD-L1^+^** | | | **CD8^+^FOXP3^+^** | | |
| --- | --- | --- | --- | --- | --- | --- | --- | --- | --- | --- | --- | --- |
|  | **High** | **Low** | **X^2^, p value** | **High** | **Low** | **X^2^, p value** | **High** | **Low** | **X^2^, p value** | **High** | **Low** | **X^2^, p value** |
| **Age** |  |  | 1.04, 0.307 |  |  | 0.14, 0.708 |  |  | 0.12, 0.733 |  |  | 3.41, 0.065 |
| < 55 | 42 | 49 |  | 48 | 44 |  | 44 | 46 |  | 24 | 16 |  |
| ≥ 55 | 67 | 59 |  | 63 | 64 |  | 60 | 57 |  | 22 | 32 |  |
| **Size** |  |  | 0.17, 0.681 |  |  | <0.01, 0.961 |  |  | 0.05, 0.829 |  |  | 1.56, 0.212 |
| ≤ 20mm | 55 | 51 |  | 50 | 49 |  | 48 | 46 |  | 27 | 22 |  |
| > 20mm | 54 | 56 |  | 61 | 59 |  | 55 | 56 |  | 19 | 26 |  |
| **LN status** |  |  | 0.67, 0.413 |  |  | 0.04, 0.848 |  |  | 0.12,0.732 |  |  | 1.22, 0.270 |
| Negative | 74 | 67 |  | 71 | 69 |  | 66 | 63 |  | 33 | 28 |  |
| Positive | 34 | 39 |  | 39 | 40 |  | 37 | 39 |  | 13 | 18 |  |
| **Chemotherapy** |  |  | 0.02, 0.879 |  |  | 1.64, 0.200 |  |  | 2.49, 0.115 |  |  | 1.15, 0.284 |
| Yes | 82 | 78 |  | 85 | 71 |  | 84 | 71 |  | 36 | 34 |  |
| No | 24 | 24 |  | 23 | 29 |  | 17 | 25 |  | 8 | 13 |  |
| **TILs** |  |  | 7.02, 0.008 |  |  | 13.26, <0.001 |  |  | 23.00, <0.001 |  |  | 3.51, 0.061 |
| < 30% | 46 | 65 |  | 42 | 68 |  | 33 | 67 |  | 17 | 27 |  |
| ≥ 30% | 63 | 43 |  | 69 | 41 |  | 71 | 36 |  | 29 | 21 |  |
| **Histology** |  |  | 0.08, 0.775 |  |  | 4.03, 0.045 |  |  | 2.50, 0.114 |  |  | 1.53, 0.216 |
| IDC | 102 | 100 |  | 106 | 96 |  | 99 | 92 |  | 41 | 46 |  |
| Other | 7 | 8 |  | 5 | 13 |  | 5 | 11 |  | 5 | 2 |  |

**Supplementary Table S2E.** **X^2^** analysis for stromal double marker phenotypes.

|  | **CD3^+^PD-1^+^** | | | **CD3^+^PD-L1^+^** | | | **FOXP3^+^PD-1^+^** | | | **CD68^+^PD-1^+^** | | |
| --- | --- | --- | --- | --- | --- | --- | --- | --- | --- | --- | --- | --- |
|  | **High** | **Low** | **X^2^, p value** | **High** | **Low** | **X^2^, p value** | **High** | **Low** | **X^2^, p value** | **High** | **Low** | **X^2^, p value** |
| **Age** |  |  | 0.36, 0.547 |  |  | <0.01, 0.971 |  |  | 0.51, 0.476 |  |  | 1.68, 0.195 |
| < 55 | 53 | 48 |  | 45 | 43 |  | 36 | 29 |  | 58 | 26 |  |
| ≥ 55 | 65 | 69 |  | 58 | 56 |  | 49 | 31 |  | 71 | 47 |  |
| **Size** |  |  | 0.16, 0.689 |  |  | 0.31, 0.579 |  |  | 2.40, 0.121 |  |  | <0.01, 0.993 |
| ≤ 20mm | 57 | 53 |  | 48 | 50 |  | 46 | 25 |  | 60 | 34 |  |
| > 20mm | 61 | 63 |  | 55 | 49 |  | 38 | 35 |  | 69 | 39 |  |
| **LN status** |  |  | 0.83, 0.363 |  |  | 0.19, 0.662 |  |  | 2.12, 0.146 |  |  | 10.55, 0.001 |
| Negative | 72 | 78 |  | 65 | 66 |  | 59 | 34 |  | 72 | 58 |  |
| Positive | 45 | 38 |  | 37 | 33 |  | 26 | 25 |  | 55 | 15 |  |
| **Chemotherapy** |  |  | 6.48, 0.011 |  |  | 2.09, 0.148 |  |  | 1.14, 0.286 |  |  | 1.48, 0.224 |
| Yes | 93 | 75 |  | 81 | 68 |  | 63 | 48 |  | 97 | 50 |  |
| No | 20 | 36 |  | 18 | 25 |  | 19 | 9 |  | 27 | 21 |  |
| **TILs** |  |  | 36.03, <0.001 |  |  | 40.78, <0.001 |  |  | 0.01, 0.916 |  |  | 2.87, 0.091 |
| < 30% | 40 | 86 |  | 26 | 70 |  | 39 | 27 |  | 60 | 43 |  |
| ≥ 30% | 78 | 32 |  | 77 | 30 |  | 46 | 33 |  | 69 | 30 |  |
| **Histology** |  |  | 7.87, 0.005 |  |  | 0.42, 0.517 |  |  | 0.26, 0.613 |  |  | 0.79, 0.378 |
| IDC | 114 | 102 |  | 98 | 93 |  | 79 | 57 |  | 121 | 66 |  |
| Other | 4 | 16 |  | 5 | 7 |  | 6 | 3 |  | 8 | 7 |  |

**Supplementary Table S2F. X^2^** analysis for stromal triple marker phenotypes.

|  | **CD3^+^CD8^+^PD-1^+^** | | | **CD3^+^CD8^+^PD-L1^+^** | | |
| --- | --- | --- | --- | --- | --- | --- |
|  | **High** | **Low** | **X^2^, p value** | **High** | **Low** | **X^2^, p value** |
| **Age** |  |  | 0.38, 0.540 |  |  | 0.32, 0.571 |
| < 55 | 47 | 49 |  | 41 | 43 |  |
| ≥ 55 | 69 | 61 |  | 53 | 47 |  |
| **Size** |  |  | 1.52, 0.217 |  |  | <0.01, 0.979 |
| ≤ 20mm | 58 | 46 |  | 43 | 41 |  |
| >20mm | 58 | 64 |  | 51 | 49 |  |
| **LN status** |  |  | 0.75, 0.386 |  |  | <0.01, 0.967 |
| Negative | 71 | 74 |  | 59 | 58 |  |
| Positive | 44 | 36 |  | 34 | 33 |  |
| **Chemotherapy** |  |  | 2.13, 0.144 |  |  | 1.57, 0.211 |
| Yes | 89 | 73 |  | 76 | 63 |  |
| No | 23 | 30 |  | 15 | 20 |  |
| **TILs** |  |  | 25.01, <0.001 |  |  | 21.58, <0.001 |
| < 30% | 42 | 77 |  | 25 | 55 |  |
| ≥ 30% | 74 | 34 |  | 69 | 36 |  |
| **Histology** |  |  | 3.16, 0.075 |  |  | 1.57, 0.210 |
| IDC | 110 | 98 |  | 90 | 83 |  |
| Other | 6 | 13 |  | 4 | 8 |  |

**Supplementary Table S3A. X^2^** analysis for CD68^+^PD-L1^+^ and single marker phenotypes.

|  | **CD3^+^** | | | **CD8^+^** | | | **CD20^+^** | | | **CD68^+^** | | |
| --- | --- | --- | --- | --- | --- | --- | --- | --- | --- | --- | --- | --- |
|  | **High** | **Low** | **X^2^, p value** | **High** | **Low** | **X^2^, p value** | **High** | **Low** | **X^2^, p value** | **High** | **Low** | **X^2^, p value** |
| **CD68^+^PD-L1^+^** |  |  | 32.79, <0.001 |  |  | 10.40, 0.001 |  |  | 0.75, 0.388 |  |  | 3.15, 0.076 |
| High | 82 | 34 |  | 73 | 43 |  | 60 | 54 |  | 67 | 49 |  |
| Low | 38 | 77 |  | 48 | 67 |  | 53 | 60 |  | 53 | 62 |  |
|  |  |  |  |  |  |  |  |  |  |  |  |  |
|  | ***PD-L1^+^*** | | | ***PD-1^+^*** | | | ***FOXP3^+^*** | | |  |  |  |
|  | **High** | **Low** | **X^2^, p value** | **High** | **Low** | **X^2^, p value** | **High** | **Low** | **X^2^, p value** |  |  |  |
| **CD68^+^PD-L1^+^** |  |  | 109.71, <0.001 |  |  | 44.53, <0.001 |  |  | 2.74, 0.098 |  |  |  |
| High | 101 | 15 |  | 86 | 30 |  | 62 | 45 |  |  |  |  |
| Low | 21 | 94 |  | 34 | 79 |  | 42 | 49 |  |  |  |  |

**Supplementary Table S3B. X^2^** analysis for CD68^+^PD-L1^+^ including double and triple marker phenotypes.

|  | **FOXP3^+^PD-L1^+^** | | | **FOXP3^+^PD-1^+^** | | | **CD20^+^PD-1^+^** | | | **CD20^+^PD-L1^+^** | | |
| --- | --- | --- | --- | --- | --- | --- | --- | --- | --- | --- | --- | --- |
|  | **High** | **Low** | **X^2^, p value** | **High** | **Low** | **X^2^, p value** | **High** | **Low** | **X^2^, p value** | **High** | **Low** | **X^2^, p value** |
| **CD68^+^PD-L1^+^** |  |  | 14.13, <0.001 |  |  | 0.07, 0.795 |  |  | 0.04, 0.833 |  |  | 6.45, 0.011 |
| High | 57 | 36 |  | 48 | 35 |  | 35 | 36 |  | 61 | 40 |  |
| Low | 13 | 34 |  | 36 | 24 |  | 17 | 16 |  | 12 | 22 |  |
|  |  |  |  |  |  |  |  |  |  |  |  |  |
|  | ***CD3^+^FOXP3^+^*** | | | ***CD8^+^PD-1^+^*** | | | ***CD8^+^PD-L1^+^*** | | | ***CD8^+^FOXP3^+^*** | | |
|  | **High** | **Low** | **X^2^, p value** | **High** | **Low** | **X^2^, p value** | **High** | **Low** | **X^2^, p value** | **High** | **Low** | **X^2^, p value** |
| **CD68^+^PD-L1^+^** |  |  | 20.98, <0.001 |  |  | 16.68, <0.001 |  |  | 58.89, <0.001 |  |  | 2.87, 0.090 |
| High | 74 | 38 |  | 74 | 41 |  | 86 | 29 |  | 31 | 23 |  |
| Low | 35 | 66 |  | 36 | 63 |  | 18 | 70 |  | 15 | 23 |  |
|  |  |  |  |  |  |  |  |  |  |  |  |  |
|  | ***CD3^+^PD-L1^+^*** | | | ***CD3^+^PD-1^+^*** | | | ***CD68^+^PD-1^+^*** | | |  |  |  |
|  | **High** | **Low** | **X^2^, p value** | **High** | **Low** | **X^2^, p value** | **High** | **Low** | **X^2^, p value** |  |  |  |
| **CD68^+^PD-L1^+^** |  |  | 72.62, <0.001 |  |  | 50.35, <0.001 |  |  | 4.46, 0.035 |  |  |  |
| High | 89 | 26 |  | 87 | 29 |  | 77 | 30 |  |  |  |  |
| Low | 14 | 71 |  | 31 | 80 |  | 50 | 37 |  |  |  |  |
|  |  |  |  |  |  |  |  |  |  |  |  |  |
|  | ***CD3^+^CD8^+^PD-1^+^*** | | | ***CD3^+^CD8^+^PD-L1^+^*** | | |  |  |  |  |  |  |
|  | **High** | **Low** | **X^2^, p value** | **High** | **Low** | **X^2^, p value** |  |  |  |  |  |  |
| **CD68^+^PD-L1^+^** |  |  | 46.26, <0.001 |  |  | 34.39, <0.001 |  |  |  |  |  |  |
| High | 86 | 29 |  | 78 | 37 |  |  |  |  |  |  |  |
| Low | 30 | 74 |  | 16 | 53 |  |  |  |  |  |  |  |

**Supplementary Table S4.** Median stromal cell densities for key identified single, double, and triple marker immunophenotypes (cells/1.13 mm^2^) for a normal control breast tissue sample.

|  | **Normal stroma** |
| --- | --- |
| **CD3^+^** | 47.77 |
| **CD8^+^** | 27.91 |
| **CD20^+^** | 11.18 |
| **CD68^+^** | 17.76 |
| **PD-1^+^** | 7.94 |
| **PD-L1^+^** | 106.80 |
| **FOXP3^+^** | 2.67 |
| **CD3^+^PD-1^+^** | 19.18 |
| **CD3^+^PD-L1^+^** | 14.27 |
| **CD3^+^FOXP3^+^** | 7.60 |
| **CD8^+^PD-1^+^** | 6.83 |
| **CD8^+^PD-L1^+^** | 19.63 |
| **CD20^+^PD-1^+^** | 6.33 |
| **CD20^+^PD-L1^+^** | 7.75 |
| **CD68^+^PD-1^+^** | 2.86 |
| **CD68^+^PD-L1^+^** | 8.07 |
| **FOXP3^+^PD-1^+^** | 1.00 |
| **FOXP3^+^PD-L1^+^** | 4.00 |
| **CD3^+^CD8^+^PD-1^+^** | 22.33 |
| **CD3^+^CD8^+^PD-L1^+^** | 13.60 |

**Supplementary Figure S1.** Bar chart illustrating the cell count data for PD-L1 status for all immunophenotypes in the stroma.

**Supplementary Figure S2.** Kaplan-Meier survival curves stratified by stromal PD-L1+ expression as high (red) or low (blue) A. Overall survival (OS) and B. breast cancer specific survival (BCSS) in the whole cohort; C. OS and D BCSS in those treated by chemotherapy. Insert hazard ratio (HR) and p value (Cox proportional hazards).

**
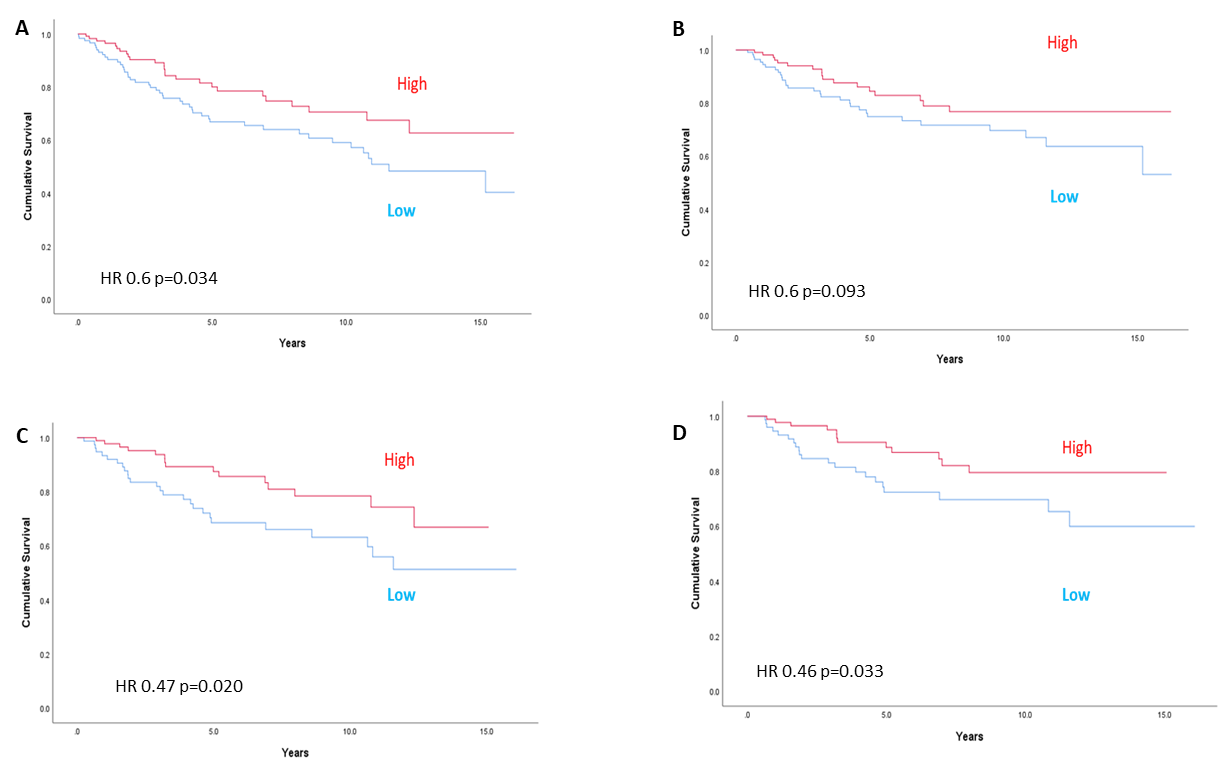
**

**Supplementary Figure S3.** Multiplexed immunofluorescence.

The individual colour channels are shown for CD8 in original pink channel (A, x400), which was re-allocated to the white channel (B, x400) for analysis to enable clear distinction from CD68; PD-L1 (C, yellow, x400) and CD68 purple channel (D, x400). The combined colour channels are presented in E. (enlarged, x400), DAPI nuclear stain blue.


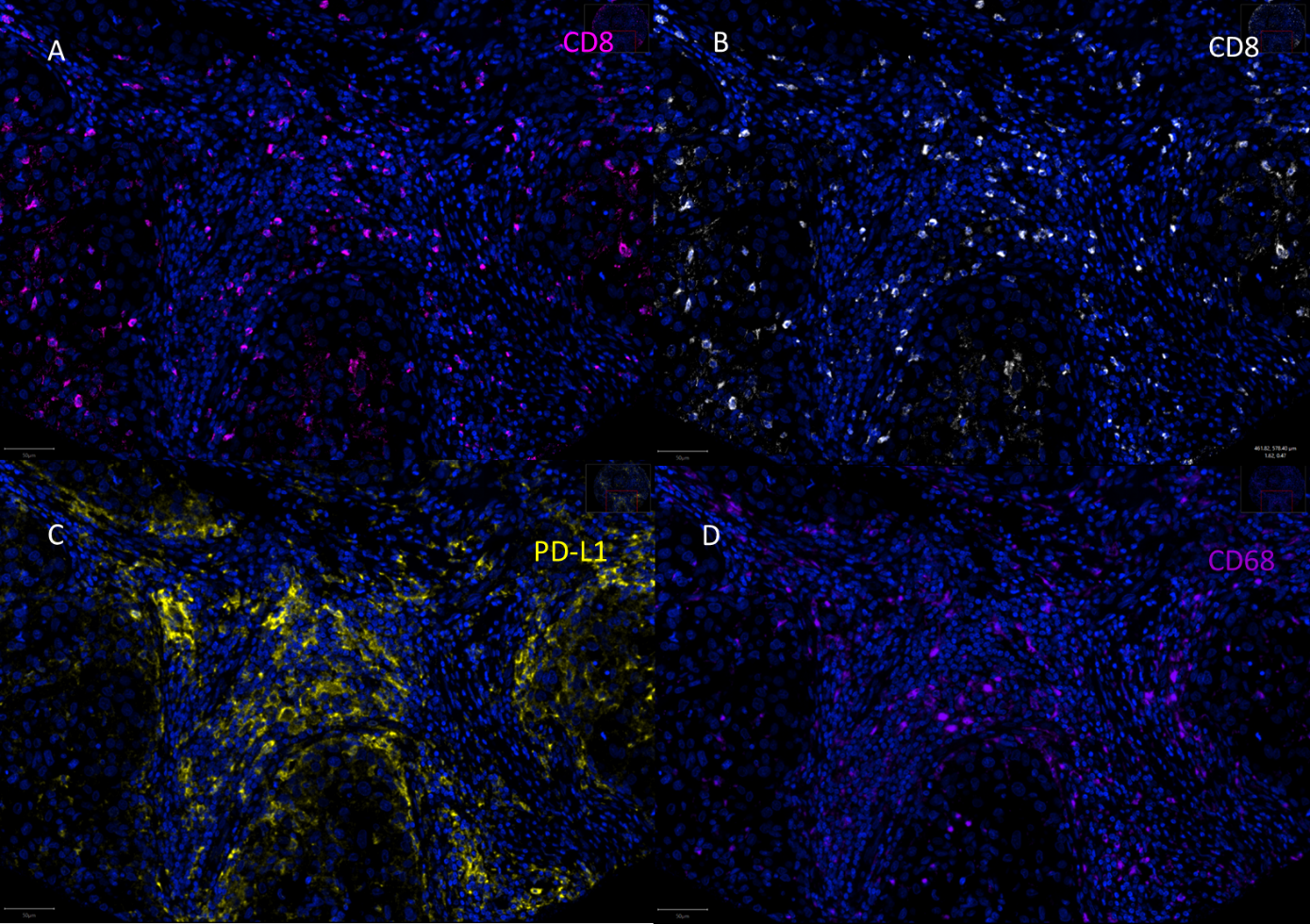


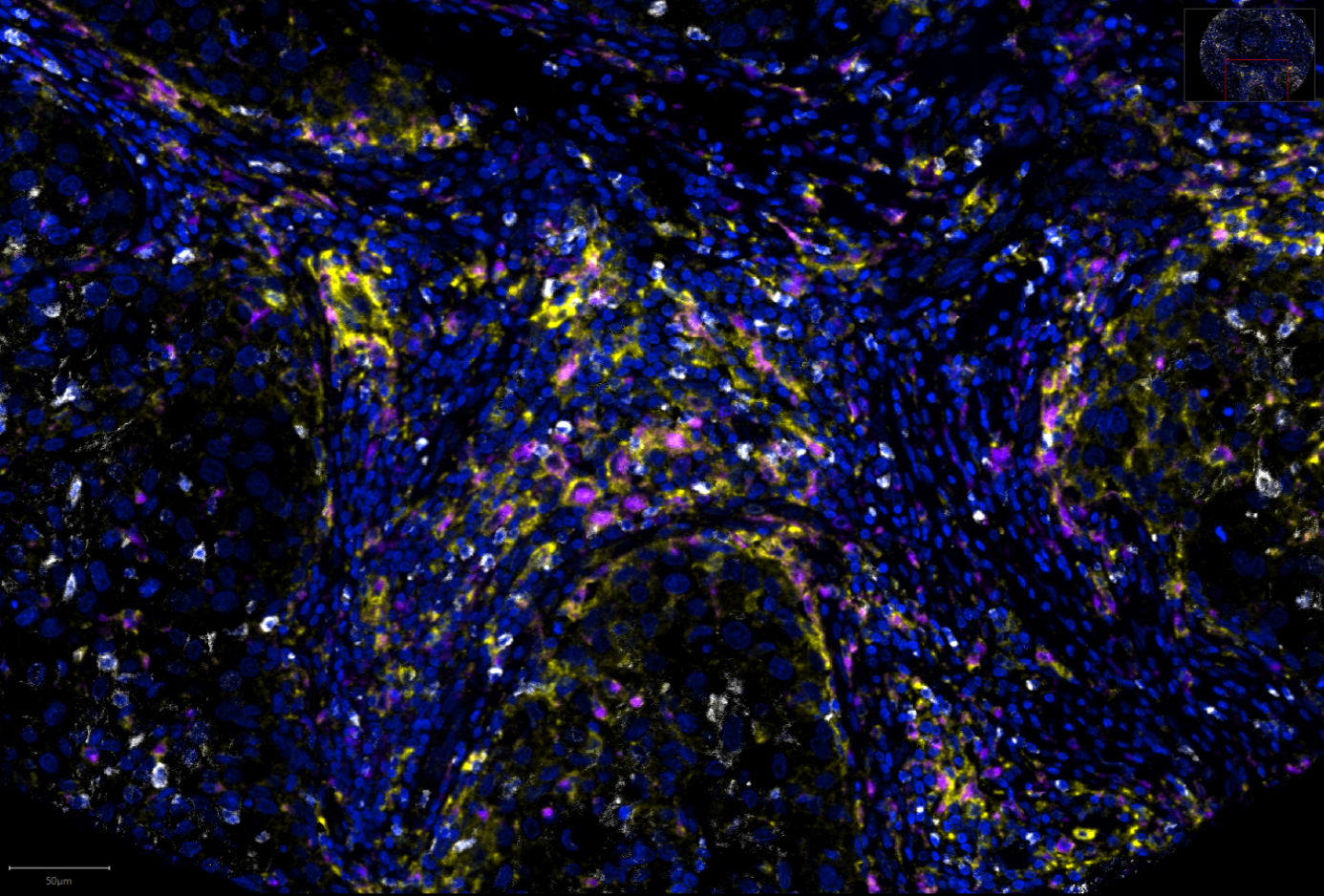


E

CD8/CD68./PD-L1
